# Comment on “Hepatic Fascioliasis Complicated by Multisite Venous Thromboembolism”: Diagnostic Challenges and Clinical Considerations

**DOI:** 10.1002/ccr3.71657

**Published:** 2025-12-09

**Authors:** Schawanya Kaewpitoon Rattanapitoon, Nathkapach Kaewpitoon Rattanapitoon

**Affiliations:** ^1^ FMC Medical Center Nakhon Ratchasima Thailand

Dear Editor,

We read with great interest the case report by Lema et al. describing *hepatic fascioliasis complicated by multisite venous thromboembolism (VTE)* published in your esteemed journal [1]. The authors presented a rare and clinically challenging scenario linking a parasitic infection with systemic thrombotic complications. While the case is intriguing and informative, we would like to offer a few constructive comments.

Firstly, the diagnosis of hepatic fascioliasis was made primarily based on imaging features and eosinophilia, supported by positive *Fasciola hepatica* IgG. However, despite repeated stool microscopy, ova were not detected. Given the known limitations of conventional stool examination during the early hepatic phase, we believe it would be valuable for the authors to clarify whether any concentration techniques or antigen detection assays were considered or available [2]. This could further strengthen the diagnostic certainty.

Secondly, the patient developed multiple thrombotic events affecting the portal, iliac, femoral veins, and pulmonary arteries. While the authors reasonably hypothesized a link between Fasciola infection and hypercoagulability, it remains unclear whether coagulation parameters beyond INR (e.g., D‐dimer, fibrinogen) were assessed. These could help to support the presence of a prothrombotic state and delineate the role of eosinophilia‐mediated endothelial injury [3].

Furthermore, anticoagulation management was complicated by the development of hepatic hematomas. Although this is a recognized complication in the hepatic migratory phase, we suggest elaborating on risk stratification strategies in future reports when initiating anticoagulation in such contexts—particularly when the diagnosis of fascioliasis is suspected but not yet confirmed [4].

Lastly, this case underscores the importance of a multidisciplinary approach, integrating infectious disease, hepatology, radiology, and hematology. We commend the authors for highlighting the need for clinical vigilance in endemic settings when eosinophilia and thrombosis coexist, even in the absence of classical symptoms.

In conclusion, this report contributes significantly to the understanding of parasite‐associated coagulopathy. Further investigation into the immunologic and coagulation mechanisms in fascioliasis‐associated VTE is warranted, and we encourage the authors to continue exploring this rare but important clinical intersection.

## Author Contributions

**Schawanya Kaewpitoon Rattanapitoon:** conceptualization, validation, visualization, writing – original draft, writing – review and editing. **Nathkapach Kaewpitoon Rattanapitoon:** supervision, validation, writing – original draft, writing – review and editing.

## Funding

The authors have nothing to report.

## Ethics Statement

The authors have nothing to report.

## Conflicts of Interest

The authors declare no conflicts of interest.

## Data Availability

All data cited are publicly available through their respective publications or databases.
